# Resilient yet productive: maize that can thrive under stress and in optimal conditions

**DOI:** 10.3389/fpls.2025.1690230

**Published:** 2025-10-13

**Authors:** Reshmi Rani Das, Madhumal Thayil Vinayan, Kaliyamoorthy Seetharam, Salahuddin Ahmad, Suriphat Thaitad, Thanh Nguyen, Manish B. Patel, Ramesh Kumar Phagna, Devraj Lenka, Pervez H. Zaidi

**Affiliations:** ^1^ Asia Maize Program, International Maize and Wheat Improvement Center (CIMMYT), Hyderabad, India; ^2^ Bangladesh Wheat & Maize Research Institute, Dinajpur, Bangladesh; ^3^ Nakhon Sawan Field Crops Research Center, Nakhon Sawan, Thailand; ^4^ National Maize Research Institute, Hanoi, Vietnam; ^5^ Anand Agricultural University, Godhra, India; ^6^ Indian Institute of Maize Research, Ludhiana, India; ^7^ Orissa University of Agriculture and Technology, Bhubaneshwar, India

**Keywords:** drought stress, rainfed maize, genotype-by-environment interaction (GEI), stress resilience, waterlogging

## Abstract

In the Asian tropics, maize is predominantly grown as a rainfed crop during the summer-rainy season, which often suffers significant yield losses due to the erratic distribution pattern of monsoon rain that causes intermittent dry spells and/or excessive moisture within the season. The climate-induced abiotic stresses, particularly drought and waterlogging, pose significant threats to rainfed maize cultivation in the Asian tropics, where erratic patterns of monsoon rain and associated high genotype-by-environment interaction (GEI) effects undermine yield stability. To address these challenges, this study evaluated 61 advanced-stage maize hybrids developed under the Asia Waterlogging and Drought Tolerant (AWDT) product profile, designed to deliver hybrids with stable grain yields under variable moisture regimes without yield penalties under optimal conditions. Multi-environment trials (METs) were conducted across 19 locations in South and Southeast Asia (India, Bangladesh, Vietnam, and Thailand) under four moisture regimes: optimal, rainfed/random stress, reproductive-stage drought, and vegetative-stage waterlogging. A stratified ranking approach was employed to identify superior hybrids that matched or exceeded commercial checks under optimal conditions and outperformed them under at least one stress environment. Several elite hybrids demonstrated broad or specific adaptation to targeted stress-prone environments. These findings underscore the importance of targeted breeding and MET-based selection strategies in developing high-performing stress-resilient maize cultivars for climate-vulnerable agroecologies, with implications for food security, farmer livelihoods, and sustainable cropping systems in the face of escalating climate variability.

## 1 Introduction

Climate change projections indicate an increased frequency of drought years, combined with heat stress and/or erratic/uneven rainfall distribution, which is likely to constrain rainfed maize production in the region severely (Tesfaye et al., 2017; Tiwari and Yadav, 2019; Singh et al., 2022). In the lowland tropics of South and Southeast Asia, the maize area expanded to over 22 million hectares (FAOSTAT, 2023). Still, yields remain below the global average, mainly because it is primarily grown as a rainfed crop, which is prone to vagaries of monsoon rains. Climate projections indicate that 20-30% of maize-growing areas in the Asian tropics are likely to experience recurrent droughts and excessive moisture, resulting in yield losses of up to 25-40% depending on stress timing and severity (Zaidi et al., 2020; Huang et al., 2022). Climate model projections further indicate that, if current varieties remain in use, rainfed maize yields may decline by 3.3–6.4% by 2030 and 5.2–12.2% by 2050, while irrigated yields may decline by 3–8% by 2030 and 5–14% by 2050 (Tesfaye et al., 2017). Significant fluctuations in year-to-year and site-to-site stress profiles (Zaidi et al., 2020) are amplifying genotype-by-environment interaction (GEI) effects and threatening the sustainability of global food systems. Among others, drought and excessive soil moisture/waterlogging stress represent a significant challenge to maize production in lowland tropics, including South and Southeast Asia (Cairns and Prasanna, 2018; Zaidi et al., 2023). Escalating climate variability, compounded by intensifying abiotic stresses, is particularly problematic for Asian tropics due to their high population density, poverty, and limited adaptive capacity (Aryal et al., 2020; Mbah et al., 2022; Zaidi et al., 2023). The climate-induced abiotic stresses further undermine genotype stability and reduce the efficiency of breeding programs that rely on consistent phenotypic selection across environments (Ceccarelli, 2015). Therefore, breeding for abiotic stress resilience in field crops is no longer a niche endeavor but a priority for food and nutritional security, which not only can help in yield stability but also improves system resilience, farmer income, and dietary outcomes (Prasanna et al., 2021). Stress-resilient crop varieties offer a vital option for risk mitigation and livelihood sustainability for millions of smallholder farmers living in marginal, stress-prone ecologies (Thomas et al., 2019; Dar et al., 2021).

In Asian tropics, maize is grown mainly as a rainfed crop, rendering it vulnerable to the erratic nature of monsoon rains and the associated abiotic and biotic constraints (Zaidi et al., 2020; Prasanna et al., 2021). The irregular distribution of monsoon rainfall contributes to the occurrence of untimely showers (Mori et al., 2021), which often result in intermittent prolonged dry spells (drought) and excessive soil moisture at different stages of crop growth during the growing season. These factors contribute to the relatively subdued productivity of maize in many parts of the Asian tropics. Additionally, maize, being a non-wetland tropical crop, shows high vulnerability to waterlogging throughout the crop cycle, particularly before tassel emergence (Zaidi et al., 2004, and Kuang et al., 2012 **).** Identification of high-performing stable maize varieties that can withstand variable weather conditions within the cropping season or have specific adaptations for a particular target population of environments (TPEs) requires a deliberate and strategic breeding and selection approach. To accomplish this, multi-environment trials (METs) are pivotal for evaluating genotypes across diverse test environments, including well-managed environments, random stress, and managed stress conditions, thereby identifying hybrids that are vulnerable to different types of stress under TPEs without yield drag under optimal conditions. The approach is a must for assessing GEI, genotypic adaptability and stability, predicting breeding values, and identifying superior genotypes with broad adaptation or suitable for specific TPE (Pour-Aboughadareh et al., 2022; Xu et al., 2022). Traditional stability parameters often undermine the complexity of GEI patterns and crossover interactions critical for stress-specific adaptation. In contrast, models like AMMI, GGE, and especially site regression (SREG) decompose the genotype main effect plus GEI into principal components, effectively distinguishing broadly adapted genotypes from those with specific adaptation (Yan and Kang, 2003; Crossa et al., 2017). By capturing both magnitude and direction of the genotypic responses, SREG facilitates precise selection across stressed and optimal environments and identifies high-performing locations with strong discriminatory power, enhancing selection efficiency and reliability. In this study, we evaluated a set of elite maize hybrids derived from the Asia waterlogging and drought-tolerant (AWDT) product profile. The breeding pipeline for this profile is designed with a renewed approach based on a different paradigm that focuses on developing maize hybrids with highly stable yields, rather than just high-yielding hybrids. The product profile aims to deliver the next generation of hybrids that achieve reasonable grain yields under adverse conditions without significantly compromising potential yields under optimal growing conditions.

## 2 Materials and methods

### 2.1 Test hybrids and testing environments

**Figure 1:**
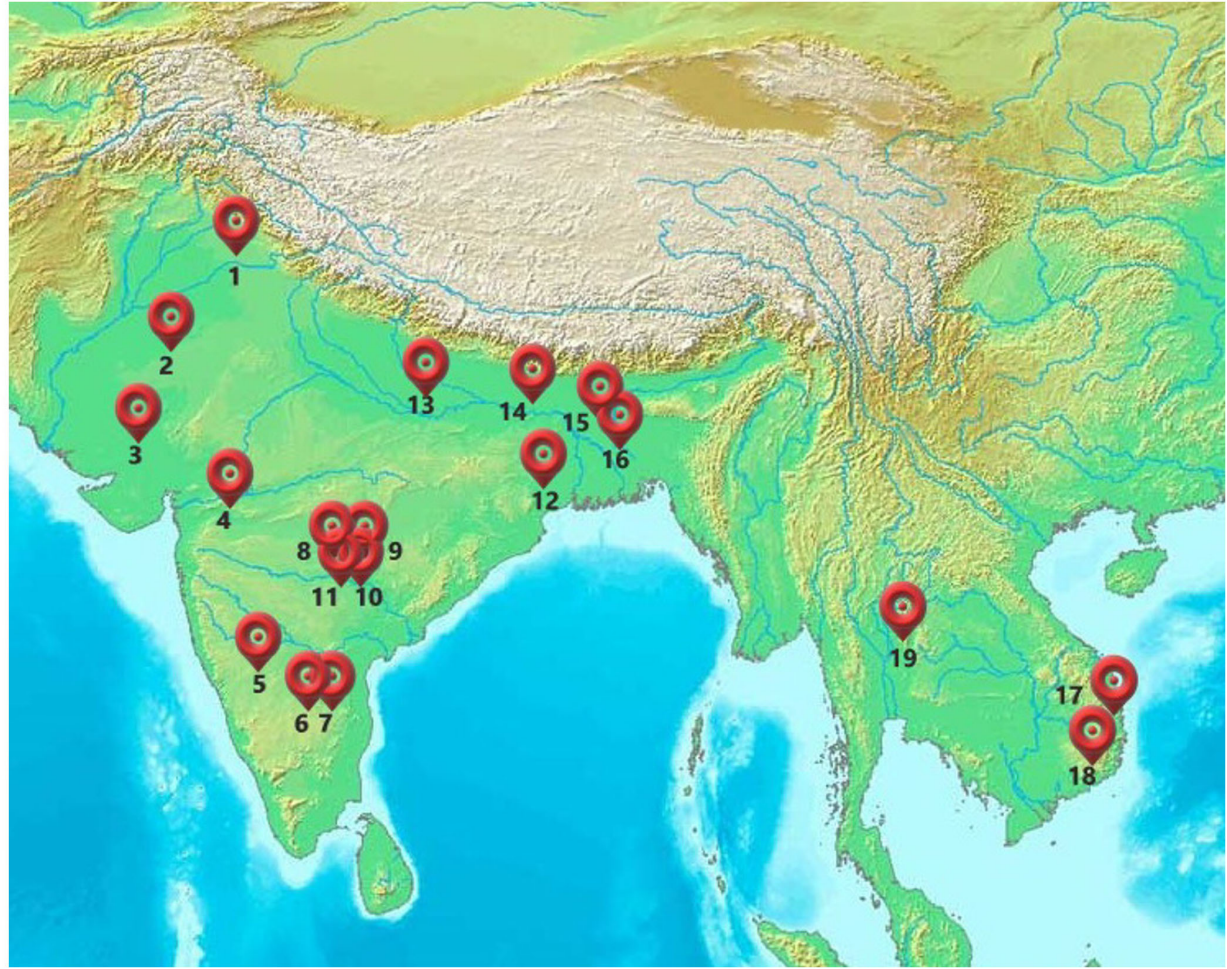
Locations of various phenotyping sites across different agroecologies in the Asian tropics. GPS coordinates of the trial locations: 1= Ludhiana (Lat-30.90,Long-75.80); 2=Chittaurgarh (Lat-24.88,Long-74.62); 3=Godhra (Lat-22.77,Long-73.61); 4=Aurangabad (Lat-19.76,Long-75.28); 5=Ranibennur (Lat-14.61,Long-75.63); 6=Bengaluru (Lat-12.97, Long-77.59); 7=Attur (Lat-13.10,Long-77.85); 8=Daulatabad (Lat-17.71,Long-78.20); 9=Yadaram (Lat-17.66,Long-78.57); 10=Shamirpet (Lat-17.58,Long-78.57); 11=Hyderabad (Lat-17.51,Long-78.27); 12=Bhubaneswar (Lat-20.26,Long-85.81); 13=Varanasi (Lat-25.26, Long-82.99); 14=Begusarai (Lat-25.41,Long-86.12); 15=Kushtia (Lat-23.89,Long-89.10); 16=Bittipara (Lat-23.79,Long-89.11); 17=Nhatrang (Lat-12.23,Long-109.19); 18=Ninh Thuan (Lat-11.67,Long-108.86); 19=Tak Fa (Lat-15.10,Long-100.38).

A set of 61 advanced-stage maize hybrids, along with two internal and two commercial check hybrids (**Supplementary Table 1**), were evaluated across multiple 19 locations in South and Southeast Asia, including India, Bangladesh, Vietnam, and Thailand (**Figure 1**). The internal checks represent CIMMYT genetics with combined drought and excessive moisture tolerance, which have been officially released and commercialized by partners in South Asia. The two commercial checks (names are intentionally not disclosed) were popular hybrids released and commercialized by multinational seed companies for cultivation during the summer rainy season in the respective TPEs. The candidate hybrids were Stage-3 hybrids from the Asia Waterlogging and Drought Tolerant (AWDT) product pipeline. These hybrids were advanced through stage-gate selection under optimal conditions and managed drought and waterlogging stresses. The test hybrids were developed under one of the CIMMYT’s maize program product profiles, namely Asia Waterlogging and Drought Tolerant (AWDT) maize, designed for the rainfed climate-vulnerable agroecologies with medium (800—1200 mm) but erratic rainfall distribution. This target population of environment (TPE) accounts for approximately 38% of the total maize area in the region. Further details of the AWDT product profile are described elsewhere (Prasanna et al., 2022). In brief, the breeding pipeline for this product profile was designed using a selected set of eight lines with good combining ability for drought and/or waterlogging tolerance and resistance to the common foliar diseases prevalent in the TPEs (*Turcicum* leaf blight and Polysora rust).

### 2.2 Trial management

The trials were constituted following an ALPHA-lattice design with two replications (Vivek et al., 2007) and planted at a spacing of 75 cm between the rows and 20 cm within rows. The trials were subjected to four different types of moisture regimes, including 1) Optimal conditions; 2) Rainfed/random stress, 3) Reproductive stage drought stress; and 4) Vegetative waterlogging stress. The optimal management trials were conducted at six sites: Bengaluru and Begusarai in India, Kushtia and Bittipara in Bangladesh, and one site each in Thailand (Tak Fa) and Vietnam (Ninh Thuan). All the trials were conducted during the summer-rainy (monsoon) season, except in Bangladesh, where the evaluations were done during the pre-monsoon season (February to May). All recommended agronomic and cultural practices were followed, including need-based supplemental irrigations in case of dry spells during the season to avoid any moisture stress. Rainfed trials were also conducted during the rainy season at four locations (Ludhiana, Chittaurgarh, Shamirpet, and Attur) under completely rainfed conditions, without any supplemental irrigation, except one irrigation immediately after planting to ensure proper seed germination and seedling establishment. Site-specific crop management practices were followed, depending on rainfall and field moisture conditions. The trials were intentionally exposed to random moisture stresses due to the erratic pattern of monsoon rains, a common phenomenon in the Asian tropics.

Managed drought stress trials were planted during the rain-free dry season (post-rainy season) at carefully selected phenotyping sites (Hyderabad and Godhra in India, and Nhatrang in Vietnam) where the winter season temperatures are generally favorable for optimal crop growth and development. Drought stress was imposed at the reproductive stage following the standardized phenotyping protocol for field drought trials (Zaman-Allah et al., 2016; Zaidi, 2019). The trials were managed with the recommended irrigation schedule until approximately two weeks before flowering. Once the accumulated growing degree days (∑GDD) reached 550 ○C, the last irrigation was applied using a high-riser sprinkler irrigation system to ensure uniform moisture across the field. The GDD was calculated using the formula below:


$$\text{Growing degree days }\left(\text{GDD}\right)=\sum \left(\frac{T_{\text{max}}+T_{\text{min}}}{2}\right)-T_{\text{base}}$$

*where*, T_max_ = maximum temperature, T_min_ = minimum temperature, and T_base_ = base temperature (10 °C).

The progress of drought stress development in the field was monitored using soil moisture profile probes placed in each of the experimental blocks across the field. Once the moisture at 40–60 cm of soil depth approached the permanent wilting point (PWP), stress was terminated by resuming irrigation. Thereafter, the required moisture level was maintained in the field to facilitate kernel development and seed setting. Waterlogging trials were conducted during the rainy season at carefully selected four precision phenotyping sites in India - Hyderabad, Begusarai, Varanasi, and Bhubaneswar, where zero-level field plots were explicitly designed for managed waterlogging stress experiments, equipped with good irrigation and drainage systems. Waterlogging stress was applied by flooding the field at the knee-high stage (V_5_-V_6_ growth stage), with a water depth of 10 ± 0.5cm maintained continuously for seven days. Water supply was monitored to ensure that it exceeded the water loss due to infiltration and evaporation. After completion of the stress treatment, the field was drained out, and subsequent moisture level was managed in the field as per the recommendations for maize crops (Zaidi et al., 2016).

**Figure 2:**
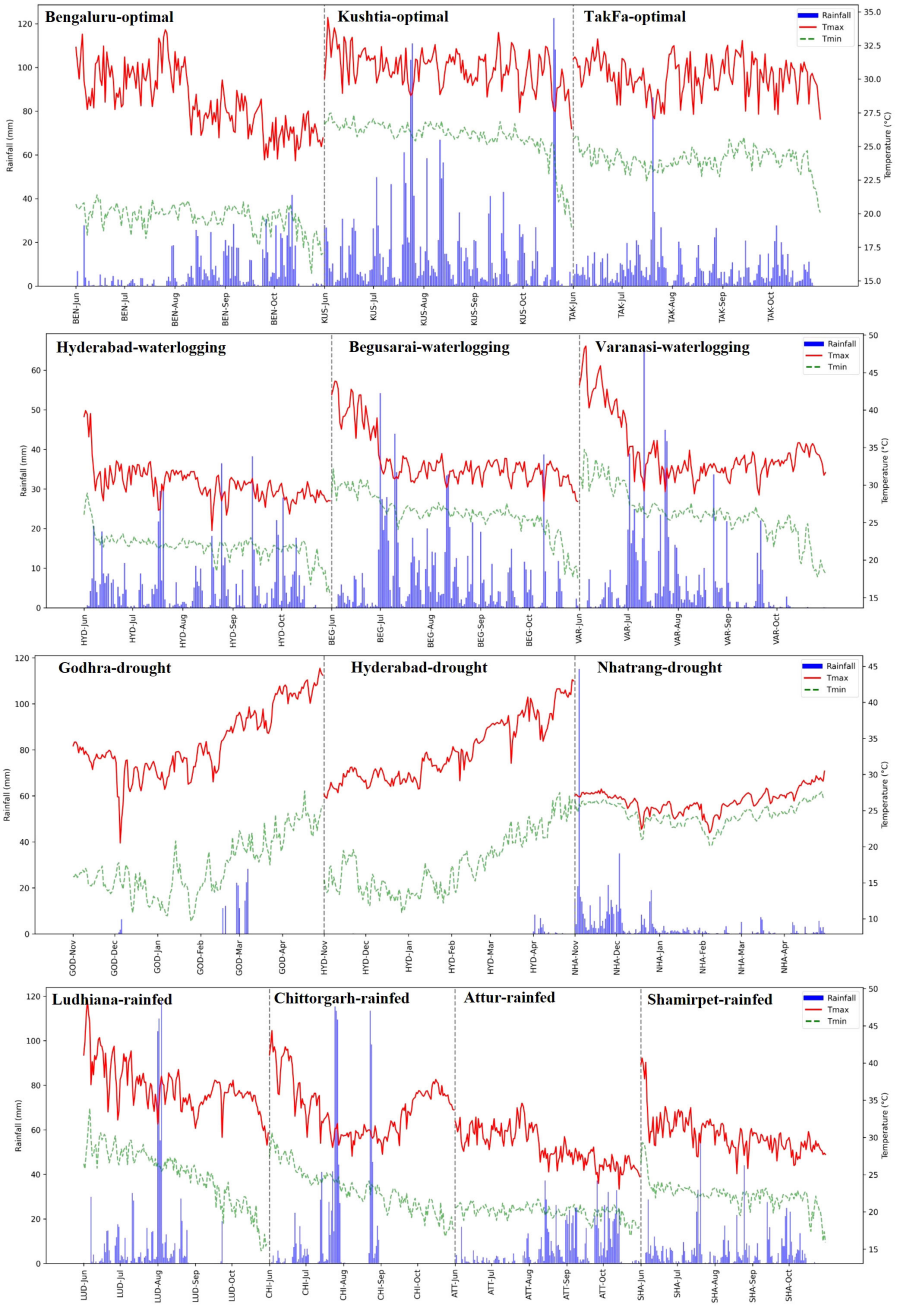
The prevailing weather conditions, including maximum and minimum temperatures and rainfall, at different locations during experiments.

Despite well-planned trials under different management conditions, some deviations occurred in managed stress and rainfed trials. For instance, the stress level was relatively moderate in managed drought trials at Nhatrang and Godhra sites due to unexpected rainfall during the trial period (**Figure 2**). Suboptimal stress management also occurred in the waterlogging trials at the Hyderabad and Varanasi sites due to unforeseen subsoil drainage issues. Similarly, the rainfed trials at Shamirpet, Attur, and Ludhiana sites in India received well-distributed rains during the season, with no exposure to random moisture stress, like intermittent drought or excessive moisture. Based on review of the location-wise weather data, the heritability and location mean for grain yield, data from seven locations (Aurangabad, Ninh Thuan, Bittipara, Ranibennur, Daulatabad, Yadaram, and Bhubaneswar) were excluded from the final analysis. The remaining 14 locations were broadly categorized into three types of environments: optimal conditions, moderate stress, and severe stress.

### 2.3 Data collection and analysis

At harvest, cob fresh weight data from each plot were recorded and grain yield (t ha^-1^) was estimated at 12.5% moisture using the following formula (ASTM, 2001):


$$\text{Grain Yield }\left(t\;ha^{-1}\right)=\frac{Cob\;weight\;\left(kg/plot\right)\times 10\times \left(\;100\;-MC\right)\times SH}{\left(100\;-\;12.5\right)\times \;Plot\;area\;\left(m^{2}\right)}$$

where MC = moisture content at harvest, SH = shelling percentage (standard 80%),

The single-site trial dataset was analyzed using the residual maximum likelihood (REML) approach (Smyth and Verbyla, 1996), treating replications and entries as random effects. Single location repeatability of the trials was computed using the genotypic variance estimates (σ2g) and single location residual (σ2ϵ) as follows:


$$\omega^{2}=\sigma^{2}g/\left[\sigma^{2}g+\sigma^{2}\epsilon \right]$$

Trials with good repeatability (ω2> 0.50) were considered in the across-location analysis and variance components estimated within each year using the following model:


$$y_{ijklm}=u+g_{i}+e_{j}+ge_{ij}+r_{k}\left(e_{j}\right)+b_{l}{\left[re\right]}_{kj}+\epsilon_{jklm}$$

where *u* denotes the overall mean; *g_i_* the genetic effect of genotype *i*; *e_j_* the effect of location *j*; *ge_ij_*, the interaction between genotype *i* and location *j*; *r_k_(e_j_)* the effect of the replication *k* nested in the location *j*; *b_l_[re]kj* the incomplete block *l* nested in the replication *k* and location *j*; and *ϵ_jklm_* the residual effect of the plot *m* nested in block *l*, replication *k*, and location *j*. In the combined analysis for each environmental group, all factors were treated as random effects.

The heritability (H) for grain yield for each environmental group was determined as follows:


$$H=\sigma^{2}g/\left[\sigma^{2}g+\left(\sigma^{2}ge/e\right)+\left(\sigma^{2}\epsilon /er\right)\right]$$

where *e* denotes the number of locations and *r* the number of replicates, *σ^2^g* is the genotypic variance, *σ^2^ge* is the variance due to genotype x environment, and *σ^2^ϵ* is the residual variance. Residuals from REML and ANOVA were checked for normality and equal variance, and PCA assumptions were verified to ensure reliable analysis of genotype performance.

Performance of the selected best-performing hybrids under each of the three distinct growing conditions— severe stress (managed drought or waterlogging stress), moderate stress (rainfed and few managed drought or waterlogging stresses with low stress intensity) and optimal environments was compared to the mean of the commercial checks using the Welch-t test for unequal sample size and unequal variances (McGee, 2025). This analysis was conducted to evaluate genotype × environment interaction (GEI) effects, including both crossover and/or non-crossover GEIs.

The software GEA-R was used to estimate the associated linear regression genotypic stability (Tai, 1971) and to assess the performance stability of genotypes across diverse environments (Angela et al., 2016). Site regression analysis was conducted using GGE model (Genotype Main Effect plus Genotype Environment Interaction), which evaluates the contribution of genotypes (G) and Genotype × Environment interaction (GEI) across diverse environments (Crossa and Cornelius, 1997), The model used for the analysis is as follows:


$$Y_{ij}=\;\mu +e_{j\;}+\sum_{n=1}^{N}\tau_{n}\gamma_{in}\delta_{jn}+\epsilon_{ij}$$

where, $Y_{ij}$ is the yield of the *i-th* genotype (i=1,.,I) in the *j-th* environment (j=1,.,J); μ is the grand mean; $e_{j\;}$ are the environmental deviations from the grand mean; $\tau_{n}$ is the eigenvalue of the PC analysis axis n; $\gamma_{in}$ and $\delta_{jn}$ are the genotype and environment principal components scores for axis n; N is the number of principal components retained in the model, and $\epsilon_{ij}$ is the error term.

The best performing hybrids across diverse stressed and unstressed environments were selected based on their performance compared to mean of the best commercial check hybrids, i.e. a selected hybrid performed - a) at least *at par* under optimal conditions, and b) *at par* moderate stress or significantly better under severe stress environments (drought or waterlogging stress). Given the high GEI effects and significant variation in mean yield of the trials across different locations, a stratified ranking approach (Kang, 1988) was employed to identify high-yielding stress-resilient hybrids that performed well under optimal conditions, and in at least one stressed environment. The data were organized in a tabular format with hybrids as rows and environments as columns. A heatmap was generated using Python (Seaborn and Matplotlib libraries) to visualize hybrid performance across environments.

## 3 Results and discussion

Grain yield is complex in inheritance, and is greatly influenced by numerous genes interacting with diverse environmental conditions. Under abiotic stresses, it typically shows low heritability and high environmental variance (Bolaños and Edmeades, 1993; Banziger et al., 2006; Araus et al., 2018). Furthermore, the relationship between grain yield under stress and non-stress conditions is often variable, unpredictable, and lacks consistency (Amadu et al., 2025; Das et al., 2020, Das et al., 2021). In this study, we evaluated a set of advanced-stage maize hybrids across a diverse range of environments, including optimal, moderate stress, and severe stress conditions, to identify hybrids with either broad adaptation (consistent performance across growing conditions) or specific adaptation (outperforming in specific growing conditions). The key findings of our study are, presented and discussed in the following sections.

### 3.1 Mean performance of hybrids under diverse environments

A comprehensive summary of statistical parameters derived from multi-location trials across diverse environments is presented in **Table 1**. Data on grain yield under different growing conditions were analyzed, and the five sites with poor data quality were discarded. The remaining 14 sites were grouped into three broad categories based on the prevailing weather conditions/stresses (**Figure 2**) and location means for grain yield (**Table 1**): severe stress (<3.0 t ha^-1^), moderate stress (3.0-6.0 t ha^-1^), and optimal environments (>6.0 t ha^-1^). The substantial genetic variability and high heritability across different locations under optimal conditions generally show higher mean performance and moderate to high heritability. Trials under severe stress also exhibit significant genotype variation, indicating the good potential for breeding stress-resilient maize hybrids.

**Table 1:** Descriptive statistics of grain yield (t ha^-1^) of advanced-stage maize hybrid trials evaluated across a range of environments in South and Southeast Asia.

Environments	Severe stress		Moderate stress					Optimal conditions						
	Beg-WL	Hyd-DT	Hyd-WL	Var-WL	God-DT	Nht-DT	Chi-RF	Sha-RF	Att-RF	Lud-RF	Kus-OP	Beg-OP	TaF-OP	Ben-OP
Location means	1.45	2.21	3.41	4.05	4.49	5.32	5.42	6.15	6.28	6.55	7.15	7.99	8.58	11.70
L.S.D.	0.51	0.86	0.52	0.35	1.51	1.18	0.88	0.76	1.22	0.71	1.02	1.12	0.99	0.90
Significance	***	***	***	**	**	***	**	**	**	**	*	*	**	*
Heritability (H)	0.71	0.74	0.79	0.73	0.89	0.62	0.68	0.48	0.59	0.81	0.95	0.44	0.79	0.82
Genotype variance	1.08	1.16	1.40	0.55	1.20	0.83	0.38	0.42	0.31	0.80	0.34	0.39	0.88	1.07
Residual variance	0.25	0.18	0.76	0.40	0.54	1.03	0.35	0.89	1.83	0.37	0.25	0.99	0.47	1.35

***, **, * refers statistical significance at p ≤ 0.001, p≤ 0.01, and p≤ 0.05 probability level, respectively.

WL, waterlogging; DT, drought; RF, rainfed; OP, optimal moisture.

Beg-WL, Begusarai (waterlogging); Nht-DT, Nhatrang (drought); Hyd-DT, Hyderabad (drought); God-DT, Godhra (drought); Hyd-WL, Hyderabad (waterlogging); Var-WL, Varanasi (waterlogging); Lud-RF, Ludhiana (rainfed); Chi-RF, Chittaurgarh (rainfed); Sha-RF, Shamirpet (rainfed); Att-RF, Attur (rainfed); TaF-OP, Tak Fa (optimal); Kus-OP, Kushtia (optimal); Ben-OP, Bengaluru (optimal.

The genotypic variation was highly significant (P<0.001) at two severe stressed (Beg-WL and Hyd-DT) and two moderate stress sites (Hyd-WL and Nht-DT). High genotype variation for grain yield under stress environments indicates that stress conditions have significantly influenced the expression of yield potential, likely due to an exacerbated GEI effects (Challinor et al., 2014; Singamsetti et al., 2022; Ljubičić et al., 2023). In stressful environments, the intense selection pressure often favors only a few resilient genotypes, resulting in lower average yields (Cortés, 2024), resulting in lower mean of the trial. The gap between the best and worst entries was relatively wide, with some entries producing almost no grain under severe stress. In essence, the inherent genetic potential of most entries over others may become less evident under severe stress conditions.

Under optimal conditions, the genetic variation was significant but relatively smaller (p<0.01 or 0.05), as all the test entries were advanced-stage hybrids already selected based on good performance during previous stage of testing in stage-gate advancement process. These findings support earlier reports that the heritability of grain yield under optimal conditions is relatively high, while it is usually low under stress (Banziger et al., 2000; Araus et al., 2018). Heritability values indicated moderate to high genetic control across environments, with Kus-OP showing the highest heritability (H = 0.95), suggesting strong potential for selection. High genotype variance at the managed drought stress site (Godhra), with lowest residual variance in the same environment, further confirmed the reliability of the site in identifying stress-resilient hybrids. These findings emphasize the importance of MET, especially in managed stress environments, to identify high-yielding maize hybrids with consistent performance. The data also highlights environments with high discriminatory power and genetic variability, which are critical for breeding programs focused on high yield and resilience (Elakhdar et al., 2025).

### 3.2 Variance components across diverse environments

The analysis of variance (ANOVA), presented in **Table 2** revealed the relative contributions of environmental (ENV), genotypic (GEN), and genotype-by-environment (GEN×ENV) interaction effects to the total variation in yield under optimal conditions, moderate stress, and severe stress conditions. Environmental factors explained a substantial proportion of the total variation, particularly under optimal conditions (64.89%), indicating that the testing locations were quite different from each other. The EVN still played a significant role under moderate and severe stress as well, highlighting local conditions strongly influence on the performance of genotypes. However, relatively lower environmental impact under stress conditions might be related to the similar level stress adverse effects imposed in the stress trials across locations, whether managed or naturally occurring stress under rainfed conditions (Bhadmus et al., 2021). Genotypic variance significantly contributed to yield variation across all environments, with the highest impact observed under severe stress, followed by optimal and moderate stress conditions. These results suggest that genetic variation among hybrids were more pronounced under stress, offering better scope for selection and breeding for stress resilience (Noor et al., 2019; Mohanapriya et al., 2023; Marid and Argaw, 2023). A significant genetic variability for grain yield across diverse environments is critical (Yue et al., 2022; Swarup et al., 2021), as it enables the identification of hybrids with either broad or specific adaptability (Derera et al., 2008).

**Table 2:** Analysis of the partitioning of the percent variation explained by different factors in the performance of genotypes across environments.

Environment	Factor	DF (factor)	SS (factor)	MS (factor)	Accumulated %age	DF (residual)	SS (residual)	MS (residual)	F-value	P-value
Optimal	ENV	6	3365.260	560.877	64.893	506.00	502.250	0.993	565.064	0.000
Moderate stress		4	418.830	104.708	29.151	339.00	237.930	0.702	149.186	0.000
Severe stress		1	42.300	42.300	26.655	149.00	44.940	0.302	140.247	0.000
Optimal	GEN	74	594.240	8.030	76.352	506.00	502.250	0.993	8.090	0.000
Moderate stress		74	215.990	2.919	44.184	339.00	237.930	0.702	4.159	0.000
Severe stress		74	84.720	1.145	80.045	149.00	44.940	0.302	3.796	0.000
Optimal	ENV*GEN	444	1226.370	2.762	100.000	506.00	502.250	0.993	2.783	0.000
Moderate stress		296	801.940	2.709	100.000	339.00	237.930	0.702	3.860	0.000
Severe stress		74	31.660	0.428	100.000	149.00	44.940	0.302	1.419	0.037
Optimal	PC1	79	819.080	10.368	43.239	506.00	502.250	0.993	10.446	0.000
Moderate stress		77	576.150	7.482	53.542	339.00	237.930	0.702	10.661	0.000
Severe stress		74	83.950	1.134	72.266	149.00	44.940	0.302	3.761	0.000
Optimal	PC2	77	435.250	5.653	66.215	506.00	502.250	0.993	5.695	0.000
Moderate stress		75	222.520	2.967	74.221	339.00	237.930	0.702	4.227	0.000
Severe stress		72	32.220	0.448	100.000	149.00	44.940	0.302	1.484	0.023
Optimal	PC3	75	209.990	2.800	77.300	506.00	502.250	0.993	2.821	0.000
Moderate stress		73	128.740	1.764	86.185	339.00	237.930	0.702	2.513	0.000

While understanding the individual contribution of genotype and environment in the performance of genotype is important, the role of GEI effects is even more critical, as it can eventually determine the final performance of a genotype within specific environments (specific adaptation) or across multiple environments (broad adaptation). In this study, the estimates of GEI variance were significantly higher than genotypic or environment variance. The GEI effects accounted for the residual variation across all environmental regimes (**Table 2**), emphasizing the dependence of genotype performance on environmental variability. This highlights the dominant role of GEI in METs and its importance in identifying adaptable genotypes (Olivoto et al., 2019; Yue et al., 2022; Argaw et al., 2025). Our results showed that 60-70% of the total yield variation across diverse environments was explained by the combined effects of environment and GEI variance. This indicates that hybrid performance varied considerably across environments, reinforcing the importance for high genetic diversity in the selection of superior hybrids (Oliveira et al., 2014; Singamsetti et al., 2023).

Site regression (SREG) analysis showed that the first principal component axis (PC1), which captures the main effect of genotype and its interaction with the environment, explained a large proportion of variation in grain yield under severe stress, followed by moderate stress and optimal conditions. The second principal component (PC2) showed a similar pattern, accounting for 100% of the GGE sum of squares under severe stress, 74.22% under moderate stress and 66.21% under optimal conditions. Interestingly, the variation caused by the environment × genotype (ENV × GEN) interaction was even greater than what was explained by PC1 and PC2. Both PC1 and PC2 had highly significant mean square (P<0.001), indicating that they are reliable indicators for the genotype expression across different environments. These results support earlier studies, which suggested that grain yields vary depending on their environmental conditions (Peiffer et al., 2014; Shrestha et al., 2021).

The complex interplay between genetic traits and environmental factors resulted in GEI effects and variability in phenotypic expression. This is reflected in the inconsistent performance and/or rankings of genotypes under different environments, making it challenging to select best performing hybrids across multiple target environments (Vinayan et al., 2020; Cooper and Messina, 2023). Overall, the results suggest that a few components can effectively capture genotype performance patterns, especially under stress, thus can facilitate genotype classification and selection (Crossa, 1990). Additionally, the residual variance was lowest under severe stress, indicating that the statistical model used in our study was accurate and reliable for managed stress environments.

### 3.3 Biplot insights into environment-specific genotype adaptation

**Figure 3:**
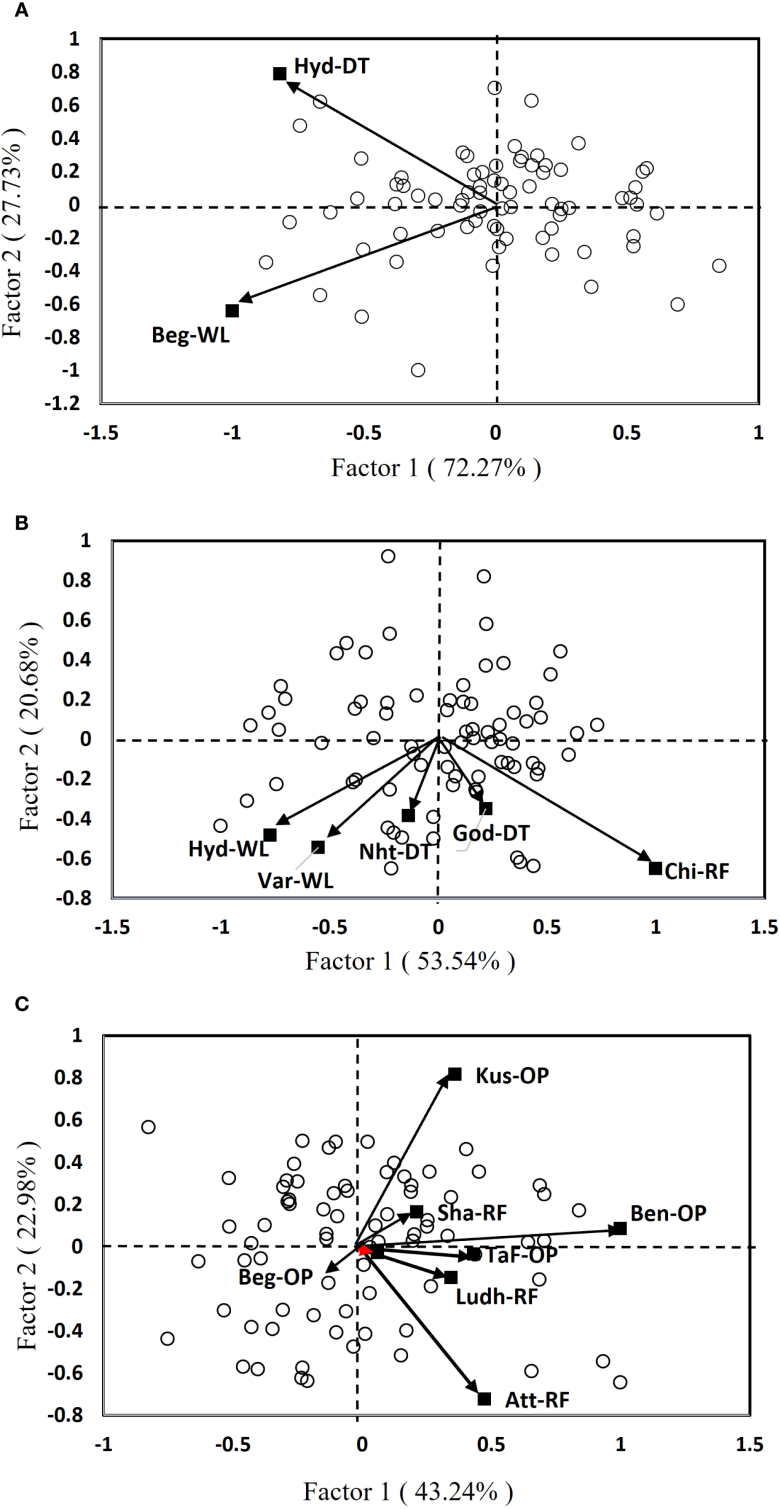
SREG bi-plots depicting the testing environments and performance of genotypes in different environments: **(A)** severe stress, **(B)** moderate stress, and **(C)** optimal environment. Beg-WL, Begusarai (waterlogging); Nht-DT, Nhatrang (drought); Hyd-DT, Hyderabad (drought); God-DT, Godhra (drought); Hyd-WL, Hyderabad (waterlogging); Var-WL, Varanasi (waterlogging); Lud-RF, Ludhiana (rainfed); Chi-RF, Chittaurgarh (rainfed); Sha-RF, Shamirpet (rainfed); Att-RF, Attur (rainfed); TaF-OP, Tak Fa (optimal); Kus-OP, Kushtia (optimal); Ben-OP, Bengaluru (optimal).

A standard GGE biplot, based on the site regression (SREG) model, was developed to assess the performance of test hybrids across stressed and unstressed environments. The first two principal components (PC1 and PC2) were obtained by singular-value decomposition of the environment-centered data (**Figure 3**). The two-dimensional graphical display enables for a visual interpretation of both genotype effects and GEI (Yan and Kang, 2003; Crossa et al., 2017). In environments with severe stress, the biplot clearly shows segregation among location and genotypes, indicating strong differential responses under stress-prone conditions (**Figure 3A**). Notably, the two locations, one with managed drought and the other with managed waterlogging stress, showed no correlation, indicating a strong crossover interaction. The high proportion of variation explained by PC1 suggests that the primary source of GEI was consistent across these stress-prone environments. Genotypes were widely dispersed on the biplot, which further confirms strong differential responses to the two abiotic stresses. In such environments, traits such as drought and/or waterlogging tolerance become crucial, and genotypes with specific adaptation to these stresses performed better, leading to crossover effects. The SREG model effectively captures these complex interactions, helping breeders identify genotypes that are well-suited to specific stress conditions (Yan and Kang, 2003).

Under moderate stress, the distribution of genotypes indicates an intermediate level of stability and performance, with some genotypes showing specific adaptation (**Figure 3B**). The relatively lower variance explained by the first two factors suggests a more complex GEI, likely influenced by intermediate stress levels or inconsistent management practices. Genotypes show moderate dispersion that reflects partial stability alongside specific adaptation. Environmental clustering is less pronounced, implying moderate correlations among locations. Notably, Hyd-WL and Var-WL sites exhibited a strong positive correlation. This suggests that these two locations share a similar stress profile (i.e., moderate waterlogging stress), and therefore a minimum possibility of crossover GEI effects. Such environments often represent transitional zones, where genotypes must balance stress tolerance with yield potential, therefore, breeding strategies should focus on general adaptability while maintaining resilience to occasional stress events (Crossa et al., 2017).

In an optimal environment, the clustering of genotypes and environments indicates consistent performance and minimum crossover interactions (**Figure 3C**). Genotypes and environments were more closely grouped, indicating high stability and low crossover interaction. Few genotypes consistently outperformed others in those favorable environments, making them strong candidates for targeting such favorable mega-environments. In both moderate stress and optimal environmental conditions, the test locations were grouped into two distinct clusters. The locations such as Kus-OP, Att-OP, and Ben-OP under optimal conditions and Chi-RF under moderate stress had longer vector in the biplot, suggesting that they were specifically useful for identifying genotypes with broad adaptability. These highly discriminating locations help breeders to select promising hybrids that perform well across TPEs (Patne et al., 2025). Under severe stress, the two principal components (PC1 and PC2) together explained 100% of the total variability in the hybrids, showing that the SREG model was highly effective in capturing how genetics and environment affect yield. Similarly, under moderate stress, the model explained 74.22% of the variation, and under optimal conditions, it explained 66.22%.

The study found that severe stress environments with low-yielding conditions were most effective in distinguishing genotypic responses to stress, followed by moderate stress and then optimal conditions. In these harsh environments, the genetic potential of genotypes is explicitly expressed in terms of their ability to adapt to adverse environments (Malenica et al., 2021, and Saad-Allah et al., 2022). Previous research has also shown that other environmental factors, beyond the main stress being studied, can also significantly affect the performance of entries (Romay et al., 2010). Often, breeding designs overlook these factors when evaluating entries and selecting test locations. Therefore, it is crucial to evaluate test genotypes in environments that truly represent the challenges of TPEs to ensure selection of suitable genotypes with high and consistent performance. By removing less informative locations in MLTs and focusing on the most representative ones, it is possible to pinpoint the most suitable hybrids while saving resources and improving genetic gains (Singamsetti et al., 2021, Singamsetti et al., 2022; Patne et al., 2025).

### 3.4 Selection of high-performing hybrids for diverse TPEs

Selections of maize hybrids based MLTs conducted under well-managed, on-station conditions can work well for favorable environments. However, this approach may fall short in climate-vulnerable regions with variable weather patterns, such as areas prone to intermittent drought and/or excessive moisture stress (Castleberry et al., 1984; Martinez-Barajas et al., 1992). Given the weather variability within and across seasons/years in the tropics, high yielding cultivars must have a high stability across a wide range of environmental conditions (Makumbi et al., 2015; Mebratu et al., 2019; Zaidi et al., 2020). In our study, we selected top-performing hybrids for diverse environmental conditions by comparing their average grain yield across locations within each type of environment compared with the average yield of the best commercial check hybrid.

**Figure 4:**
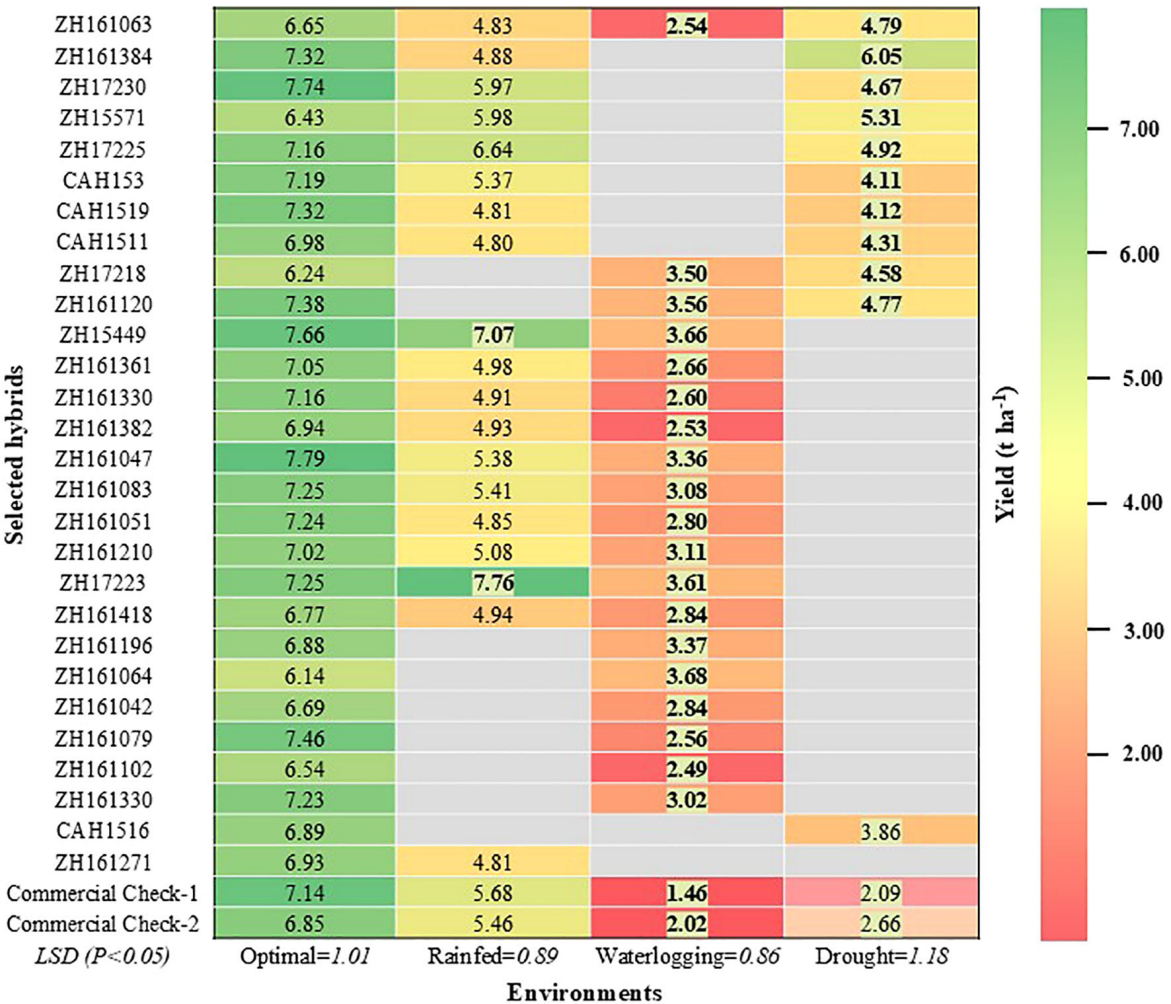
Heat map depicting the relative performance of selected best hybrids across different environments compared to the mean of the best commercial checks. The yield values in bold and shaded fonts indicate significantly better performance, and those in regular font performed *at par* with the best commercial check.

The results showed significant variation in hybrid performance across environments, highlighting the importance of specific adaptation. Among the 28 selected hybrids, only one hybrid (ZH161063) was able to perform well across the diverse types of stresses as well as optimal conditions (**Figure 4**). We also identified several hybrids with targeted stress tolerance, including 10 hybrids tolerant to waterlogging and rainfed/random stress, 7 hybrids tolerant to drought and rainfed/random stress, and two hybrids tolerant to both drought and waterlogging stress. These hybrids combined high yield potential with resilience, making them strong candidates for deployment in diverse agroecologies. Earlier studies by Zaidi et al. (2008), Zaidi et al. (2010) demonstrated the relationship between drought and excess moisture tolerance and proposed the selection criteria with desirable traits for combined stress tolerance. Additionally, two hybrids were tolerant to only one type of stress, i.e., either drought or waterlogging stress. These findings align with a recent multi-country study by Tarekegne et al. (2024), which reported annual grain yield gains of 118 kg ha^-^¹ under optimal conditions and 61 kg ha^-^¹ under random stress, demonstrating that simultaneous improvement under both conditions is achievable.

The superior performance of the top hybrids under drought stress can be attributed to a combination of physiological and biochemical traits. These include better water-use efficiency and deeper, more robust root systems that enhance drought resilience (Zaidi et al., 2003; Cairns et al., 2013; Zaidi et al., 2022). Under waterlogged conditions, the development of adventitious roots, formation of aerenchyma (air spaces in roots), and efficient anaerobic metabolism help the plant survive in low-oxygen environments. Additionally, the ability to maintain photosynthesis during short-term stress supports overall plant health and yield (Zaidi et al., 2007; Arora et al., 2017; Liang et al., 2020). These traits collectively enhance oxygen transport and root survival, thereby contributing to improved tolerance against waterlogging stress. Our study demonstrated the value of testing hybrids across wide range of environments to identify promising hybrids with either broad or specific adaptations. The observed GEIs are consistent with the recent studies that emphasize the role of physiological traits and genomic selection in enhancing maize performance under abiotic stress (Amadu et al., 2025).

The strong performance of certain hybrids in stress-prone environments indicates that a targeted product pipeline approach (such as AWDT in CIMMYT) for climate-vulnerable agroecologies has successfully introduced adaptive traits. Our findings are consistent with recent progress in tropical maize breeding, where multi-trait genomic prediction models are used to improve yield, drought tolerance, and disease resistance simultaneously (Prasanna et al., 2021). Including local checks hybrids in the trials provides a benchmark, and the consistent outperformance by elite hybrids validates the genetic gains achieved through AWDT breeding pipeline of CIMMYT’s Asia maize program (Prasanna et al., 2022). Recent research emphasizes the importance of trait plasticity, a ability of genotype in response to environmental changes, as a key strategy for maintaining consistent performance across diverse conditions. Chapman et al. (2021) argued that selecting for plasticity, rather than fixed tolerance traits, can improve both yield stability and adaptability across stress and non-stress environments. Together, these studies (Banziger et al., 2006; Zaidi et al., 2020; Singamsetti et al., 2021; Prasanna et al., 2022) challenge the long-held belief that breeding for stress resilience inevitably results in a yield penalty under optimal conditions. Instead, they show that resilience and high productivity can be achieved simultaneously.

## 4 Conclusion

The study revealed significant genetic variability among test hybrids developed through the AWDT product pipeline, suggesting strong potential for selecting high-performing stress-resilient hybrids. The GEI effects accounted for 60-70% of yield variation, highlighting the strong influence of environmental conditions in hybrid performance. Site regression (SREG) analysis demonstrated that severe stress environments offered the greatest ability to distinguish among genotypes, making them ideal for identifying superior stress-tolerant hybrids. In contrast, under moderate stress and optimal conditions, genotypes exhibited greater stability. Additionally, the test locations are grouped into distinct clusters, helping identify environments best suited for genotype evaluation.

Several hybrids met CIMMYT’s criteria, designed for smallholder farmers in stress-prone agroecologies of South and Southeast Asia. These hybrids consistently outperformed the standard check hybrids, demonstrating strong potential for future commercialization. The selected high-yielding, stress-resilient hybrids are being advanced through the stage-gate advancement process for large-scale on-farm testing across stress-prone, rainfed agroecologies in the tropical regions of Asia. Our findings highlight the importance of using strategic, targeted product pipeline-based breeding approach, such as AWDT profile,—in breeding programs aiming at rainfed climate vulnerable region in Asian lowland tropics. Across location testing in high-discriminating environments can enhance cost-efficiency and ensure selection of maize hybrids that sustain high yields under both stressed and favorable conditions. This approach supports climate-adaptive agriculture for marginal, stress-prone ecologies in lowland tropical regions. The results advocate for a shift in breeding strategy: moving from a focus solely on maximizing yield under favorable conditions to also minimizing risk under stress, while maintaining acceptable yield levels. This paradigm enhances the resilience, productivity, and sustainability of maize-based farming systems in vulnerable tropical agroecologies.

## Data Availability

The original contributions presented in the study are included in the article/**Supplementary Material**. Further inquiries can be directed to the corresponding author.

## References

[B1] Amadu M. K. Beyene Y. Chaikam V. Tongoona P. B. Danquah E. Y. Ifie B. E.. (2025). Genome-wide association mapping and genomic prediction analyses reveal the genetic architecture of grain yield and agronomic traits under drought and optimum conditions in maize. BMC Plant Biol. 25, 135. doi: 10.1186/s12870-025-06135-3, PMID: 39893411 PMC11786572

[B2] Angela P. Mateo V. Gregorio A. Francisco R. Marco L. Jose C.. (2016). GEA-R (Genotype environment analysis with R for windows), version 4.0 (El-Batán, Mexico: CIMMYT).

[B3] Araus J. L. Kefauver S. C. Zaman-Allah M. Olsen M. S. Cairns J. E. (2018). Translating high-throughput phenotyping into genetic gain. Trends Plant Sci. 23, 451–466. doi: 10.1016/j.tplants.2018.02.001, PMID: 29555431 PMC5931794

[B4] Argaw T. Fenta B. A. Zegeye H. Azmach G. Funga A. (2025). Multi-environment trials data analysis: linear mixed model-based approaches using spatial and factor analytic models. Front. Res. Metr. Anal. 10. doi: 10.3389/frma.2025.1472282, PMID: 40297568 PMC12034942

[B5] Arora K. Panda K. K. Mittal S. Mallikarjuna M. G. Rao A. R. Dash P. K.. (2017). RNAseq revealed the important gene pathways controlling adaptive mechanisms under waterlogged stress in maize. Sci. Rep. 7, 10950. doi: 10.1038/s41598-017-10561-1, PMID: 28887464 PMC5591269

[B6] Aryal J. P. Sapkota T. B. Khurana R. Khatri-Chhetri A. Rahut D. B. Jat M. L. (2020). Climate change and agriculture in South Asia: adaptation options in smallholder production systems. Environ. Dev. Sustain 22, 5045–5075. doi: 10.1007/s10668-019-00414-4

[B7] Banziger M. Edmeades G. O. Beck D. Bellon M. (2000). “Breeding for drought and low nitrogen tolerance,” in From theory to practice (CIMMYT, Mexico, D.F).

[B8] Banziger M. Setimela P. S. Hodson D. Vivek B. (2006). Breeding for improved abiotic stress tolerance in maize adapted to southern Africa. Agric. Water Manage. 80, 212–224. doi: 10.1016/j.agwat.2005.07.014

[B9] Bhadmus O. A. Badu-Apraku B. Adeyemo O. A. Ogunkanmi A. L. (2021). Genetic analysis of early white quality protein maize inbreds and derived hybrids under low-nitrogen and combined drought and heat stress environments. Plants 10, 2596. doi: 10.3390/plants10122596, PMID: 34961067 PMC8706249

[B10] Bolaños J. Edmeades G. O. (1993). Eight cycles of selection for drought tolerance in lowland tropical maize. I. Responses in grain yield, biomass, and radiation utilization. Field Crops Res. 31, 233–252. doi: 10.1016/0378-4290(93)90064-T

[B11] Cairns J. E. Crossa J. Zaidi P. H. Grudloyma P. Sanchez C. Araus J. L.. (2013). Identification of drought, heat and combined drought and heat tolerance donors in maize (Zea mays L.). Crop Sci. 53 (4), 1335–1346. doi: 10.2135/cropsci2012.09.0545

[B12] Cairns J. E. Prasanna B. (2018). Developing and deploying climate-resilient maize varieties in the developing world. Curr. Opin. Plant Biol. 45, 226–230. doi: 10.1016/j.pbi.2018.05.004, PMID: 29779966 PMC6250980

[B13] Castleberry R. M. Crum C. W. Krull C. F. (1984). Genetic yield improvement of U.S. Maize cultivars under varying fertility and climatic environments. Crop Sci 24, cropsci1984.0011183X002400010008x. doi: 10.2135/cropsci1984.0011183X002400010008x

[B14] Ceccarelli S. (2015). Efficiency of plant breeding. Crop Sci 55, 87–97. doi: 10.2135/cropsci2014.02.0158

[B15] Challinor A. J. Watson J. Lobell D. B. Howden S. M. Smith D. R. Chhetri N. (2014). A meta-analysis of crop yield under climate change and adaptation. Nat. Clim Change 4, 287–291. doi: 10.1038/nclimate2153

[B16] Chapman S. Birch C. E. Galdos M. V. Pope E. Davie J. Bradshaw C.. (2021). Assessing the impact of climate change on soil erosion in East Africa using a convection-permitting climate model. Environ. Res. Lett. 16, 084006. doi: 10.1088/1748-9326/ac10e1

[B17] Cooper M. Messina C. D. (2023). Breeding crops for drought-affected environments and improved climate resilience. Plant Cell 35, 162–186. doi: 10.1093/plcell/koac321, PMID: 36370076 PMC9806606

[B18] Cortés A. J. (2024). Abiotic stress tolerance boosted by genetic diversity in plants. Int. J. Mol. Sci. 25, 5367. doi: 10.3390/ijms25105367, PMID: 38791404 PMC11121514

[B19] Crossa J. (1990). “Statistical analyses of multilocation trials,” in Advances in agronomy. Ed. Brady N. C. (Washington, DC: Science and Technology, Agency for International Development, Department of State), 40, 55–85. doi: 10.1016/S0065-2113(08)

[B20] Crossa J. Cornelius P. L. (1997). Sites regression and shifted multiplicative model clustering of cultivar trial sites under heterogeneity of error variances. Crop Sci 37, 406–415. doi: 10.2135/cropsci1997.0011183x003700020017x

[B21] Crossa J. Pérez-Rodríguez P. Cuevas J. Montesinos-López O. Jarquín D. de Los Campos G.. (2017). Genomic selection in plant breeding: methods, models, and perspectives. Trends Plant Sci. 22, 961–975. doi: 10.1016/j.tplants.2017.08.011, PMID: 28965742

[B22] Dar M. H. Bano D. A. Waza S. A. Zaidi N. W. Majid A. Shikari A. B.. (2021). Abiotic stress tolerance-progress and pathways of sustainable rice production. Sustainability 13, 2078. doi: 10.3390/su13042078

[B23] Das R. R. Vinayan M. T. Patel M. B. Phagna R. K. Singh S. B. Shahi J. P.. (2020). Genetic gains with rapid-cycle genomic selection for combined drought and waterlogging tolerance in tropical maize (Zea mays L.). Plant Genome 13, e20035. doi: 10.1002/tpg2.20035, PMID: 33217198 PMC12806969

[B24] Das R. R. Vinayan M. T. Seetharam K. Patel M. Phagna R. K. Singh S. B.. (2021). Genetic gains with genomic versus phenotypic selection for drought and waterlogging tolerance in tropical maize (Zea mays L.). Crop J. 9, 1438–1448. doi: 10.1016/j.cj.2021.03.012 PMC12806969 33217198

[B25] Derera J. Tongoona P. Pixley K. V. Vivek B. Laing M. D. van Rij N. C. (2008). Gene action controlling gray leaf spot resistance in southern african maize germplasm. Crop Sci 48, 93–98. doi: 10.2135/cropsci2007.04.0185

[B26] Elakhdar A. El-Naggar A. A. El-Wakeell S. Ahmed A. H. (2025). Integrating univariate and multivariate stability indices for breeding clime-resilient barley cultivars. BMC Plant Biol. 25, 76. doi: 10.1186/s12870-024-05530-6, PMID: 39825255 PMC11748582

[B27] FAOSTAT (2023). Agricultural production, crop primary database (Rome: Food and Agriculture Organization of the United Nations). Available online at: http://www.fao.org/faostat/en/data/QC/ (Accessed September 20, 2025).

[B28] Huang C. Gao Y. Qin A. Liu Z. Zhao B. Ning D.. (2022). Effects of waterlogging at different stages and durations on maize growth and grain yields. Agric. Water Manage. 261, 107334. doi: 10.1016/j.agwat.2021.107334

[B29] Kang M. S. (1988). A rank-sum method for selecting high-yielding, sta ble corn genotypes. Cereal Res. Commun. 16, 113–115.

[B30] Kuang W. Xianjiang Y. Xiuqing C. Yafeng X. (2012). Experimental study on water production function for waterlogging stress on corn. Proc. Eng. 28, 598–603. doi: 10.1016/j.proeng.2012.01.775

[B31] Liang K. Tang K. Fang T. Qiu F. (2020). Waterlogging tolerance in maize: genetic and molecular basis. Mol. Breed. 40, 111. doi: 10.1007/s11032-020-01190-0

[B32] Ljubičić N. Popović V. Kostić M. Pajić M. Buđen M. Gligorević K.. (2023). Multivariate interaction analysis of zea mays L. Genotypes growth productivity in different environmental conditions. Plants 12, 2165. doi: 10.3390/plants12112165, PMID: 37299146 PMC10255084

[B33] Makumbi D. Diallo A. Kanampiu F. Mugo S. Karaya H. (2015). Agronomic performance and genotype × Environment interaction of herbicide-resistant maize varieties in eastern africa. Crop Sci 55, 540–555. doi: 10.2135/cropsci2014.08.0593

[B34] Malenica N. Dunić J. A. Vukadinović L. Cesar V. Šimić D. (2021). Genetic approaches to enhance multiple stress tolerance in maize. Genes 12, 1760. doi: 10.3390/genes12111760, PMID: 34828366 PMC8617808

[B35] Marid Y. Argaw T. (2023). Evaluation of maize varieties through multi-environment trials: application of multiplicative mixed models. Cornous Biol. 1, 1–12. doi: 10.37446/corbio/rsa/1.2.2023.1-12

[B36] Martinez-Barajas E. Villanueva-Verduzco C. Molina-Galán J. Loza-Tavera H. Sánchez-de-Jiménez E. (1992). Relation of rubisco to maize grain yield improvement: effect of water restriction. Crop Sci 32, 718–722. doi: 10.2135/cropsci1992.0011183X003200030028x

[B37] Mbah M. F. Shingruf A. Molthan-Hill P. (2022). Policies and practices of climate change education in South Asia: towards a support framework for an impactful climate change adaptation. Clim Action 1, 28. doi: 10.1007/s44168-022-00028-z

[B38] McGee R. W. (2025). Welch’s T-test: the robust default for comparing two groups in psychological research. Sociology Soc. Policy 2, 14–20. doi: 10.26855/ssp.2025.12.003

[B39] Mebratu A. Wegary D. Mohammed W. Teklewold A. Tarekegne A. (2019). Genotype × Environment interaction of quality protein maize hybrids under contrasting management conditions in eastern and southern africa. Crop Sci 59, 1576–1589. doi: 10.2135/cropsci2018.12.0722

[B40] Mohanapriya B. Ravikesavan R. Senthil N. Iyanar K. Senthil A. Sathya Sheela K. R. V. (2023). Genetic variation and trait association of maize hybrids under irrigated and drought-stress environments. Electron. J. Plant Breed. 13, 1343–1353. doi: 10.37992/2022.1304.178

[B41] Mori N. Takemi T. Tachikawa Y. Tatano H. Shimura T. Tanaka T.. (2021). Recent nationwide climate change impact assessments of natural hazards in Japan and East Asia. Weather Climate Extremes 32, 100309. doi: 10.1016/j.wace.2021.100309

[B42] Noor J. J. Vinayan M. T. Umar S. Devi P. Iqbal M. Zaidi K. S., P. H. (2019). Morpho-physiological traits associated with heat stress tolerance in tropical maize (Zea mays L.) at reproductive stage. Aust. J. Crop Sci. 13, 536–545. doi: 10.21475/ajcs.19.13.04.p1448

[B43] Oliveira E. J. de, Freitas J. P. X. Jesus O. N. (2014). AMMI analysis of the adaptability and yield stability of yellow passion fruit varieties. Sci. Agric. (Piracicaba Braz.) 71, 139–145. doi: 10.1590/S0103-90162014000200008 25177932

[B44] Olivoto T. Lúcio A. D. C. da Silva J. A. G. Marchioro V. S. de Souza V. Q. Jost E. (2019). Mean performance and stability in multi-environment trials I: combining features of AMMI and BLUP techniques. Agron. J. 111, 2949–2960. doi: 10.2134/agronj2019.03.0220

[B45] Patne N. Takalkar S. A. Mohan S. M. Naidu P. B. Lohithaswa C. H. Kachapur R. M.. (2025). Genotype and environmental interactions in maize (Zea mays L.) across regions of India: Implications for hybrid testing locations in South Asia. Aust. J. Crop Sci. 19, 773–783. doi: 10.21475/ajcs.25.19.07.p330

[B46] Peiffer J. A. Romay M. C. Gore M. A. Flint-Garcia S. A. Zhang Z. Millard M. J.. (2014). The genetic architecture of maize height. Genetics 196, 1337–1356. doi: 10.1534/genetics.113.159152, PMID: 24514905 PMC3982682

[B47] Pour-Aboughadareh A. Khalili M. Poczai P. Olivoto T. (2022). Stability indices to deciphering the genotype-by-environment interaction (GEI) effect: an applicable review for use in plant breeding programs. Plants 11, 414. doi: 10.3390/plants11030414, PMID: 35161396 PMC8839246

[B48] Prasanna B. M. Burgueño J. Beyene Y. Makumbi D. Asea G. Woyengo V.. (2022). Genetic trends in CIMMYT’s tropical maize breeding pipelines. Sci. Rep. 12, 20110. doi: 10.1038/s41598-022-24536-4, PMID: 36418412 PMC9684471

[B49] Prasanna B. M. Cairns J. E. Zaidi P. H. Beyene Y. Makumbi D. Gowda M.. (2021). Beat the stress: breeding for climate resilience in maize for the tropical rainfed environments. Theor. Appl. Genet. 134, 1729–1752. doi: 10.1007/s00122-021-03773-7, PMID: 33594449 PMC7885763

[B50] Romay M. C. Malvar R. A. Campo L. Alvarez A. Moreno-González J. Ordás A.. (2010). Climatic and genotypic effects for grain yield in maize under stress conditions. Crop Sci 50, 51–58. doi: 10.2135/cropsci2008.12.0695

[B51] Saad-Allah K. M. Nessem A. A. Ebrahim M. K. H. Gad D. (2022). Evaluation of drought tolerance of five maize genotypes by virtue of physiological and molecular responses. Agronomy 12, 59. doi: 10.3390/agronomy12010059

[B52] Shrestha J. Subedi S. Acharya R. Sharma S. Subedi M. (2021). Grain Yield Stability of Maize (Zea mays L.) Hybrids using Ammi Model and GGE Biplot Analysis. SAARC J. Agric. 19, 107–121. doi: 10.3329/sja.v19i2.57675

[B53] Singamsetti A. Shahi J. Zaidi P. Seetharam K. (2022). Study on applicability of genotype × yield × trait (GYT) biplots over genotype × trait (GT) biplots in selection of maize hybrids across soil moisture regimes. Indian J. Agric. Res. doi: 10.18805/ijare.a-5850

[B54] Singamsetti A. Shahi J. P. Zaidi P. H. Seetharam K. Vinayan M. T. Kumar M.. (2021). Genotype × environment interaction and selection of maize (Zea mays L.) hybrids across moisture regimes. Field Crops Res. 270, 108224. doi: 10.1016/j.fcr.2021.108224

[B55] Singamsetti A. Zaidi P. H. Seetharam K. Vinayan M. T. Olivoto T. Mahato A.. (2023). Genetic gains in tropical maize hybrids across moisture regimes with multi-trait-based index selection. Front. Plant Sci. 14. doi: 10.3389/fpls.2023.1147424, PMID: 36938016 PMC10020505

[B56] Singh G. M. Gosavi G. Patel S. S. Solanki P. S. Zhang F. Xu J.. (2022). Impact of drought on maize yield and exploration of *in-situ* maize crop genetic resources for drought tolerance. Preprints. doi: 10.20944/preprints202212.0210.v1

[B57] Smyth G. K. Verbyla A. P. (1996). A conditional likelihood approach to residual maximum likelihood estimation in generalized linear models. J. R. Stat. Society: Ser. B (Methodological) 58, 565–572.

[B58] Swarup S. Cargill E. J. Crosby K. Flagel L. Kniskern J. Glenn K. C. (2021). Genetic diversity is indispensable for plant breeding to improve crops. Crop Sci. 61, 839–852. doi: 10.1002/csc2.20377

[B59] Tai G. C. C. (1971). Genotypic stability analysis and its application to potato regional trials. Crop Sci. 11, 184–190. doi: 10.2135/cropsci1971.0011183x001100020006x

[B60] Tarekegne A. Wegary D. Cairns J. E. Zaman-Allah M. Beyene Y. Negera D.. (2024). Genetic gains in early maturing maize hybrids developed by the International Maize and Wheat Improvement Center in Southern Africa during 2000–2018. Front. Plant Sci. 14. doi: 10.3389/fpls.2023.1321308, PMID: 38293626 PMC10825029

[B61] Tesfaye K. Zaidi P. H. Gbegbelegbe S. Boeber C. Rahut D. B. Getaneh F.. (2017). Climate change impacts and potential benefits of heat-tolerant maize in South Asia. Theor. Appl. Climatol 130, 959–970. doi: 10.1007/s00704-016-1931-6

[B62] Thomas K. Hardy R. D. Lazrus H. Mendez M. Orlove B. Rivera-Collazo I.. (2019). Explaining differential vulnerability to climate change: A social science review. WIREs Climate Change 10, e565. doi: 10.1002/wcc.565, PMID: 31007726 PMC6472565

[B63] Tiwari Y. K. Yadav S. K. (2019). High temperature stress tolerance in maize (Zea mays L.): physiological and molecular mechanisms. J. Plant Biol. 62, 93–102. doi: 10.1007/s12374-018-0350-x

[B64] Vinayan M. T. Zaidi P. H. Seetharam K. Rani Das R. Viswanadh S. Ahmed S.. (2020). Genotype-by-environment interaction effects under heat stress in tropical maize. Agronomy 10, 1998. doi: 10.3390/agronomy10121998

[B65] Vivek B. S. Kasango J. Chisoro S. Magorokosho C. (2007). Field-book: Software for managing a maize breeding program: A cookbook for handling field experiments, data, stocks and pedigree information (Mexico: CIMMYT).

[B66] Xu N. Qiao Y. Zhao S. Yang X. Li J. Fok M. (2022). Optimizing the test locations and replicates in multi-environmental cotton registration trials in southern Xinjiang. China. Crop Sci. 62, 1866–1879. doi: 10.1002/csc2.20811

[B67] Yan W. Kang M. S. (2003). GGE biplot analysis: A graphical tool for breeders, geneticists, and agronomists (Boca Raton, Florida: CRC Press).

[B68] Yue H. Gauch H. G. Wei J. Xie J. Chen S. Peng H.. (2022). Genotype by environment interaction analysis for grain yield and yield components of summer maize hybrids across the huanghuaihai region in China. Agriculture 12, 602. doi: 10.3390/agriculture12050602

[B69] Zaidi P. H. (2019). Management of drought stress in field phenotyping (Mexico: CIMMYT). Available online at: https://hdl.handle.net/10883/19998.

[B70] Zaidi P. H. Maniselvan P. Yadav P. Singh A. K. Sultana R. Dureja P.. (2007). Stress-adaptive changes in tropical maize (Zea mays L.) under excessive soil moisture stress. Maydica 52, 159–171.

[B71] Zaidi P. H. Mehrajuddin M. L. Jat M. L. Pixley K. Singh R. P. Dass S. (2010). “Resilient maize for improved and s table productivity of rain-fed environment of South and South East Asia,” in Maize for asia: emerging trends and technologies. Proceedings of the 10th asian regional maize workshop. eds. P. H. Zaidi, M. Azrai, K. V. Pixley, (Mexico: CIMMYT).

[B72] Zaidi P. H. Nguyen T. Ha D. N. Thaitad S. Ahmed S. Arshad M.. (2020). Stress-resilient maize for climate-vulnerable ecologies in the Asian tropics. Aust. J. Crop Sci. 14 (08), 1264–1274. doi: 10.21475/ajcs.20.14.08.p2405

[B73] Zaidi P. H. Rafique S. Rai P. K. Singh N. N. Srinivasan G. (2004). Tolerance to excess moisture in maize (Zea mays L.): susceptible crop stages and identification of tolerant genotypes. Field Crops Res. 90, 189–202. doi: 10.1016/j.fcr.2004.03.002

[B74] Zaidi P. H. Seetharam K. Vinayan M. T. Rashid Z. Krishnamurthy L. Vivek B. S. (2022). Contribution of root system architecture and function in the performance of tropical maize (Zea mays L.) genotypes under different moisture regimes. Aust. J. Crop Sci 16, 809–818. doi: 10.21475/ajcs.22.16.06.p3572

[B75] Zaidi P. H. Srinivasan G. Sanchez C. (2003). Morpho-physiological traits associated with variable field performance of different types of maize germplasm across multiple environments. Maydica 48, 207–220.

[B76] Zaidi P. H. Vinayan. M. T. Nair S. K. Kuchanur P. H. Kumar R. Bir Singh S.. (2023). Heat-tolerant maize for rainfed hot, dry environments in the lowland tropics: From breeding to improved seed delivery. Crop J. 11, 986–1000. doi: 10.1016/j.cj.2023.06.008

[B77] Zaidi P. H. Vinayan. M. T. Seetharam K. (2016). Phenotyping for abiotic stress tolerance in maize: Waterlogging stress. A field manual (Hyderabad, India: CIMMYT).

[B78] Zaidi P. H. Yadav M. Singh D. K. Singh R. P. (2008). Relationship between drought and excess moisture tolerance in tropical maize (Zea mays L.). Aust. J. Crop Sci. 1, 78–96.

[B79] Zaman-Allah M. Zaidi P. H. Trachsel S. Cairns J. E. Vinayan M. T. Seetharam K. (2016). Phenotyping for abiotic stress tolerance in maize: Drought stress. A field manual (Mexico: CIMMYT).

